# Long noncoding RNA CERS6‐AS1 functions as a malignancy promoter in breast cancer by binding to IGF2BP3 to enhance the stability of CERS6 mRNA

**DOI:** 10.1002/cam4.2675

**Published:** 2019-11-08

**Authors:** Gang Bao, Jianjun Huang, Wei Pan, Xing Li, Tian Zhou

**Affiliations:** ^1^ Breast Surgery Affiliated Hospital of Guizhou Medical University Guiyang Guizhou China; ^2^ Inspection Institute Guizhou Medical University Guiyang Guizhou China

**Keywords:** breast cancer, CERS6, CERS6‐AS1, IGF2BP3

## Abstract

Breast cancer (BC) leads to the highest mortality in women worldwide, characterized by inevitable proliferation and metastasis of BC cells. Mounting evidence confirm that lncRNAs play a significant role in the tumorigenesis and development of BC. lncRNA CERS6‐AS1 is a novel discovery, and its role and molecular mechanism in BC has not been studied. In this study, it was discovered that CERS6‐AS1 was overexpressed in BC tissues and cells. CERS6‐AS1 accelerated cell proliferation and suppressed cell apoptosis in BC. Moreover, molecular mechanism exploration uncovered that there was a positive association between CERS6 and CERS6‐AS1 (or IGF2BP3) expression in BC. Furthermore, IGF2BP3 serves as a RNA‐binding protein for CERS6‐AS1 and CERS6‐AS1 promoted CERS6 mRNA stability by binding to IGF2BP3. In the end, rescue experiments verified that overexpression of CERS6 rescues the inhibition of CERS6‐AS1 deficiency on BC progression in vitro and vivo. Taken together, these evidences suggested that CERS6‐AS1 promoted the progression of BC by binding to IGF2BP3 and thus enhancing the stability of CERS6 mRNA, providing a new underlying therapeutic target for BC to improve prognosis.

## Graphical abstract

CERS6‐AS1 is overexpressed in BC tissues and cell lines. CERS6‐AS1 promotes proliferation and inhibits apoptosis in BC cells. CERS6‐AS1 promotes CERS6 mRNA stability by binding to IGF2BP3. CERS6‐AS1 functions as a malignancy promoter in BC by binding to IGF2BP3 to enhance the stability of CERS6 mRNA.

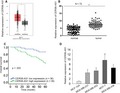

## 1 INTRODUCTION

As a main cause of cancer‐related mortality worldwide, breast cancer (BC) makes up for 6.6% of all cancer deaths. It shows the highest incidence as well as the highest mortality rate in female.^1^ Although the 5‐year survival rate of BC can reach 90%, it remarkably decreased in advanced BC, at roughly 6%.^2^ Adjuvant bisphosphonates (BPs) are recommended to many postmenopausal (PM) women as part of periodic early BC treatment. Although experiments have revealed that the prevalence of severe side effects of BPs is low, many patients were troubled by its side effect from time to time.^3^ Various biomarkers of BC have been studied,^4^ while the prognosis sustains disappointing. Hence, it is of substantial consequence to delve into the underlying regulatory mechanism of specific biomarkers in the development of BC.

Long noncoding RNA (lncRNA) is distinguished from other RNAs by its length of more than 200 nucleotides.^4^, ^5^ Aberrantly expressed lncRNAs could upregulate or downregulate some oncogenes related with cell proliferation, migration and invasion at the post‐transcriptional level.^6^ Lnc‐ASAH2B‐2a,^7^ lncRNA H19^8^ and SNHG14^9^ have been uncovered as tumor promoters in the development of BC and lncRNA FGF13‐AS1 has been discovered to suppress the occurrence of BC.^10^ Apart from sponging and sequestering specific microRNAs,^11^ lncRNAs could also modulate the expression of targeting genes via binding with RNA‐binding proteins (RBPs).^12^ LncRNA ceramide synthase 6 antisense RNA 1 (CERS6‐AS1) is a novel discovery, and its role has not studied in cancers. Thus the biological functions and molecular mechanism of CERS6‐AS1 in BC deserve exploration.

In this study, we intended to investigate whether CERS6‐AS1 acts as an oncogene in the development of BC. Finally, we came to a conclusion that CERS6‐AS1 functions as a malignancy promoter in breast cancer by binding to IGF2BP3 to enhance the stability of CERS6 mRNA.

## 2 MATERIALS AND METHODS

### 2.1 Patients and clinical samples

In this study, 72 pairs of BC tissues and paracancerous tissues were obtained from patients who experienced BC surgery at our hospital to construct tissue microarrays (TMAs). Those patients were suffered with BC between 2010 and 2014, and their tissue samples as well as detailed clinicopathologic annotation were accessible. 1.5‐mm‐diameter cores were applied to construct TMAs. These tissues were quickly frozen and then kept at −80°C. Pathologists examined the excised tissues one by one. In this study, no anti‐cancer treatment was operated on the patients before surgery. All patients signed printed informed consent. The Ethics Committee of our hospital approved this work.

### 2.2 Cell culture and transfection

BC cells (MDA‐MB‐436, MDA‐MB‐453, MCF‐7 and MDA‐MB‐231) and normal human breast cell (MCF‐10A) employed in our research were all bought from the American ATCC cell bank. MDA‐MB‐231 cells were cultured in DMEM culture medium (Thermo Fisher Scientific.) adding with 5% DMSO and 20% FBS. Other cells were placed in RPMI 1640 medium (Gibco) supplemented with 10% fetal bovine serum (FBS; Gibco/Invitrogen Inc). Cells were cultured at 37°C, with 5% CO_2_ humidified atmosphere.

The empty plasmids (mock) were obtained from GenePharma. In order to knockdown CERS6‐AS1, IGF2BP3 and CERS6 in cells, short hairpin RNA (shRNA) aiming at CERS6‐AS1, IGF2BP3 and CERS6 were designed and synthesized by GenePharma respectively. The full‐length sequences or the 3′‐UTR sequences of CERS6‐AS1, IGF2BP3 and CERS6 were, respectively, synthesized and subcloned into pcDNA3.1 vector (Invitrogen) to produce pcDNA‐CERS6‐AS1, pcDNA‐IGF2BP3 and pcDNA‐CERS6. These plasmids were transfected into MDA‐MB‐231 or MCF‐7 cells by employing Lipofectamine 2000 (Invitrogen) under the instruction of the manufacturer.

### 2.3 RNA extraction and quantitative real‐time PCR (RT‐qPCR)

Total RNA were extracted from MDA‐MB‐231 or MCF‐7 cells by utilizing Trizol reagent (Takara). RNAs were reverse transcribed into cDNA through utilizing the reverse transcription kit (Takara). The RT‐qPCR was operated by SYBR Green PCR Kit (Takara). Internal control was GAPDH. Applied Biosystems Step One Plus Real‐Time PCR System (Applied Biosystems) was employed to analyze the results of RT‐qPCR, and the 2^−ΔΔCt^ method was employed to examine these relative expression levels.

### 2.4 CCK‐8 assay

Cell Counting Kit‐8 (CCK‐8; Dojindo Molecular Technologies, Inc) was utilized to evaluate cell proliferation ability under instructions of the manufacturer. The transfected cells were seeded into 96‐well culture plates at a density of 1 × 10^3^ cells per well. Afterwards, the transfected cells underwent 24, 48, 72 and 96 hours of incubation, followed by adding CCK‐8 solution (100 μL) to each well. Then the incubation continued for another 4 hours. The absorbance at 450 nm was examined by employing a Multiskan Go spectrophotometer (Thermo Fisher Scientific, Inc).

### 2.5 Colony formation assay

Transfected cells (1 × 10^3^ cells/well) were seeded into 6‐well plates and cultured in RPMI 1640 medium. We replaced the medium every 3 days. We utilized methanol to fix colonies and 0.1% crystal violet to stain colonies after 14 days. Then, colonies were counted by experimenters.

### 2.6 Ethynyl deoxyuridine (EdU) incorporation assay

An EdU assay was conducted utilizing an EdU immunofluorescence staining kit (Ribobio). The sterilized slides were put into a 12‐well plate, and then around 1 × 10^3^ cells were seeded into each well. The transfected cells were cultured with 50 μmol/L EdU reagent for 2 hours and washed with PBS (HyClone), followed by fixation with 4% phosphate‐buffered paraformaldehyde. Afterwards the cells were stained with 100 μL of fresh Apollo reaction cocktail, and the nucleus was stained with 100 μL of Hoechst 33 342, followed by viability determination with a fluorescence microscope (Olympus BX51).

### 2.7 Determination of caspase‐3 activation

The activity of caspase‐3 was measured by the application of the cell permeable fluorogenic substrate, PhiPhiLux‐G1D2 (OncoImmunin Inc) under instructions of the manufacturer. In one group, the cells were transfected with vector and pcDNA‐CERS6‐AS1 for 24 hours, and then incubated with PhiPhiLux‐G1D2. In the other group, the cells were transfected with shCtrl, shCERS6‐AS1#1 and shCERS6‐AS1#2 for 24 hours, and then incubated with PhiPhiLux‐G1D2. The activity of caspase‐3 was detected by fluorescence microscopy.

### 2.8 Terminal deoxynucleotidyl transferase dUTP nick end labeling (TUNEL) assay

Cell apoptosis of transfected cells was analyzed by TUNEL assay. After transfection, cells were fixed in 4% (w/v) paraformaldehyde at 4°C for a quarter. TUNEL kit (Roche) was employed to examine TUNEL staining. A fluorescence microscope (Olympus) was utilized to count TUNEL‐positive cells.

### 2.9 Transwell assay

This assay was carried out employing a Transwell insert chamber coated with or without Matrigel (BD Biosciences). The cells were incubated in the upper layer containing 300 μL serum‐free medium, and the bottom chamber was filled with 10% FBS. After being cultured for 48 hours, cells left in the upper chamber were cleaned, and cells that migrated or invaded the bottom chambers were pictured and counted under a microscope.

### 2.10 Fluorescent in‐situ hybridization (FISH) assay

MDA‐MB‐231and MCF‐7 cells were fixed in 3% formaldehyde for 10 minutes and then rinsed with PBS. Pepsin (1% in 10 mmol/L HCl) was employed to operate on the fixed cells, and then ethanol was utilized to dehydrate cells repeatedly. The dehydrated cells were mixed with 40 nmol/L of the FISH probe (Hoechst, CERS6‐AS1) in a hybridization buffer and then incubated at 80°C for 2 minutes. Being left to stand at 55°C for 2 hours, the slides were then rinsed and dried, and finally observed and identified with Prolong Gold Antifade Reagent utilizing 1× Hoechst 33 342. The RNA FISH probe was provided by Ribobio.

### 2.11 Subcellular fractionation assay of RNA

In order to separate nuclear RNA from cytoplasmic RNA, PARISTM kit (Ambion, AM1921) was employed in line with the protocols of the manufacturer. U6 RNA was utilized as nuclear control and GAPDH as cytoplasmic control. Subcellular fractionation assay was performed in BC cells.

### 2.12 Western blotting

Cells were dissolved with RIPA lysis buffer (Beyotime Biotechnology) added with protease inhibitors (Roche). Thereafter, proteins were extracted. Protein concentration was examined by Enhanced BCA Protein Assay Kit (Beyotime). Proteins were isolated by SDS PAGE and then transferred onto PVDF membranes. The membranes were sealed by skim milk and then incubated with primary antibody. The primary antibody to IGF2BP3 was purchased from Abcam Company (Abcam). After being rinsed thrice, the membranes were incubated with a secondary antibody at 4°C for one night. Signals were visualized by ECL.

### 2.13 RNA pull‐down assay

RNA pull‐down assay was conducted in line with the suggestion of the manufacturer. In brief, a single biotinylated desthiobiotinylated cytidine was attached to RNA 3′ terminus by employing T4 RNA ligase. The biotinylated CERS6‐AS1 and biotinylated CERS6‐AS1 antisense were, respectively, incubated with cellular protein extracted from MDA‐MB‐231 or MCF‐7 cells, and afterwards streptavidin beads were introduced. After 48 hours, the recovered proteins associated with bio‐CERS6‐AS1 or bio‐CERS6‐AS1 antisense or control were resolved. The eluted solutions were analyzed by western blot.

### 2.14 RNA immunoprecipitation (RIP)

RIP experiments were conducted by employing the Magna RIP RNA‐Binding Protein Immunoprecipitation kit (Millipore). MDA‐MB‐231 and MCF‐7 cells were dissolved and the whole‐cell lysates were cultured with protein A+G magnetic beads, which were conjugated using IGF2BP3 antibodies (Abcam, ab26271) or control IgG at 4°C overnight. The immunoprecipitated RNA was then purified and quantified by RT‐qPCR.

### 2.15 Establishment of a stably transfected MCF‐7 cell

Stably transfected MCF‐7 cells were established as previously described.^13^ After construction of LV‐shCERS6‐AS1 or LV‐shCtrl, different concentration of puromycin was added into every group; 7 days later, these groups were observed under a microscope. The minimum lethal concentration of puromycin was applied to screen out the stably transfected MCF‐7 cell.

### 2.16 Tumorigenesis test in vivo

Male Balb/C mice, which are four‐weeks old, were bought from Shi Laike Company and reserved in our hospital with the approval of ethics committee of our hospital. Nude mice were given xenografts of sh‐CERS6‐AS1#1‐transfected, shCERS6‐AS1#1 and pcDNA‐CERS6 co‐transfected and control MCF‐7 cells (5 × 10^6^ cells per site). The mice were observed for four weeks, and then euthanatized to remove the tumors.

### 2.17 Statistical analysis

Statistical analyses were conducted with the employment of SPSS 21.0 statistical software (IBM). Data were displayed by mean ± standard deviation. Each experiment was performed thrice. Comparisons among different groups were conducted with the one‐way ANOVA or Student's *t* test. Any value of *P* < .05 was treated as statistically significant.

## 3 RESULTS

### 3.1 CERS6‐AS1 is overexpressed in BC tissues and cells

**Figure 1:**
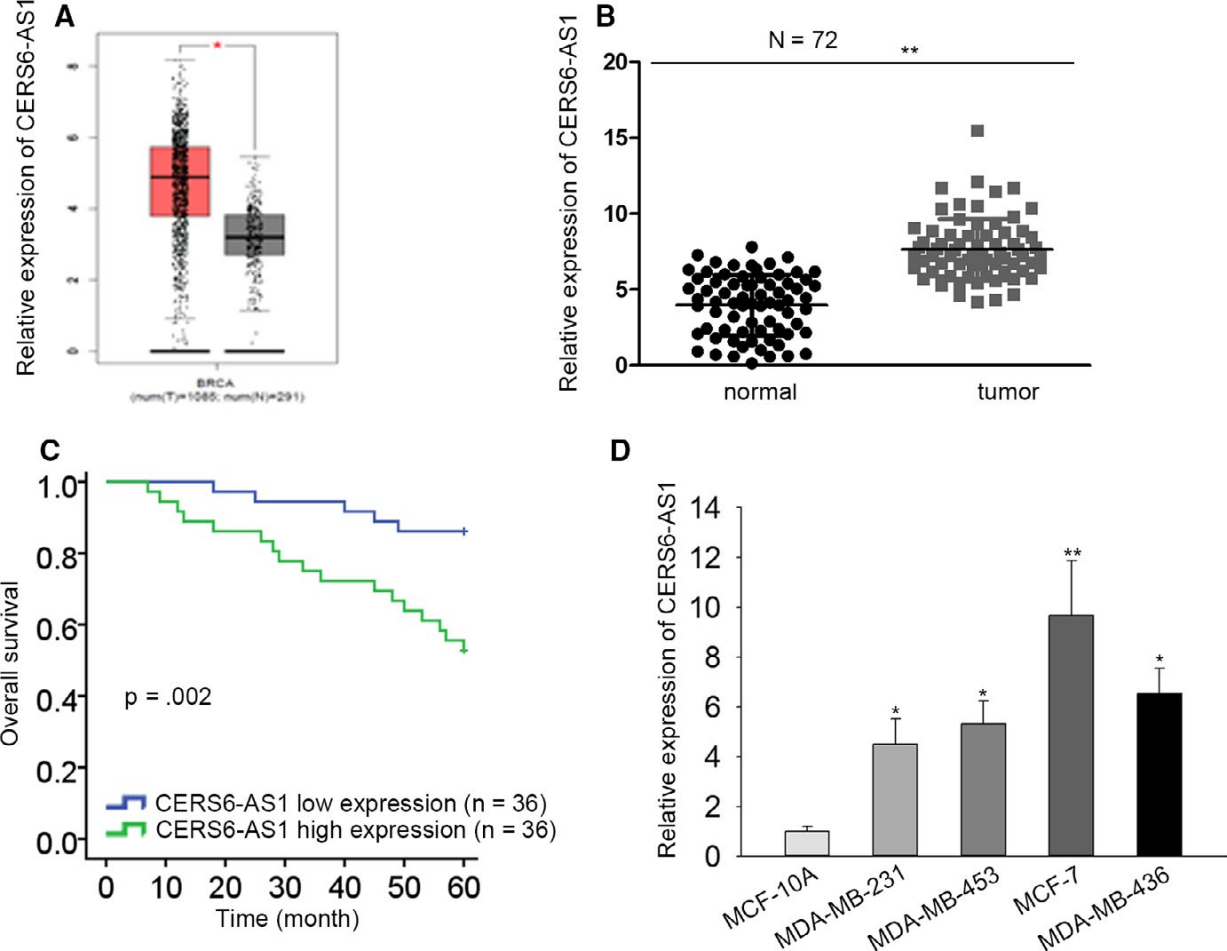
CERS6‐AS1 expression is upregulated in BC tissues and cells. A, The expression of CERS6‐AS1 in BC tissues (n = 1085) was higher than that in contiguous normal tissues (n = 291) in TCGA database. B, RT‐qPCR results revealed that expression of CERS6‐AS1 was higher in tumor tissues than in contiguous normal tissues (n = 72). C, Kaplan‐Meier survival curves described the relationship between CERS6‐AS1 expression and survival time of BC patients. D, RT‐qPCR results revealed that CERS6‐AS1 level was higher in BC cells (MDA‐MB‐436, MDA‐MB‐453, MCF‐7 and MDA‐MB‐231) than in normal human breast cells (MCF‐10A). *P < .05, **P < .01

To explore the roles of CERS6‐AS1 in BC, we obtained its expression in BC tissues and contiguous normal tissues from TCGA database (1085 tumor tissues and 291 normal tissues). The result illustrated that the CERS6‐AS1 level was dramatically upregulated in BC tissues than that in contiguous normal tissues (Figure 1A). To further confirm the upregulation of CERS6‐AS1 expression in BC, CERS6‐AS1 expression in 72 paired BC tissues and contiguous normal tissues were quantified. The result is consistent with the above‐mentioned one: the expression of CERS6‐AS1 was notably upregulated in BC tissues (Figure 1B). Moreover, it was analyzed that BC patients with high level of CERS6‐AS1 were suffered from poorer prognosis (Figure 1C). There was a remarkable increase in CERS6‐AS1 expression in BC cells (MDA‐MB‐436, MDA‐MB‐453, MCF‐7, and MDA‐MB‐231) in comparison with that in the normal human breast cells (MCF‐10A), of which CERS6‐AS1 expression was the lowest in MDA‐MB‐231 cells and the highest in MCF‐7 cells (Figure 1D). Table 1 summarized that CERS6‐AS1 level had close correlation with differentiation grade and TNM stage. Based on these findings, CERS6‐AS1 is overexpressed in BC tissues and cells.

**Table 1:** Correlation between CERS6‐AS1 expression and clinical features (n = 72)

Variable	CERS6‐AS1 Expression		*P*‐value
	low	high	
Age			
≤50	12	10	.798
>50	24	26	
Her‐2 status			
Negative	12	14	.806
Positive	24	22	
PR status			
Negative	15	13	.809
Positive	21	23	
ER status			
Negative	13	16	.631
Positive	23	20	
TNBC status			
TNBC	20	18	.814
Non‐TNBC	16	18	
Tumor size			
<20 mm	18	16	.814
≥20 mm	18	20	
Involved lymph node			
Negative	20	9	.156*
Positive	16	27	
Differentiation grade			
Well & Moderate	21	10	.017*
Poor	15	26	
TNM stage			
I/II	23	12	.018*
III/IV	13	24	

Note

Low/high by the sample median. Pearson χ^2^ test.

* *P* < .05 was considered to be statistically significant.

### 3.2 CERS6‐AS1 promotes proliferation and inhibits apoptosis in BC cells

**Figure 2:**
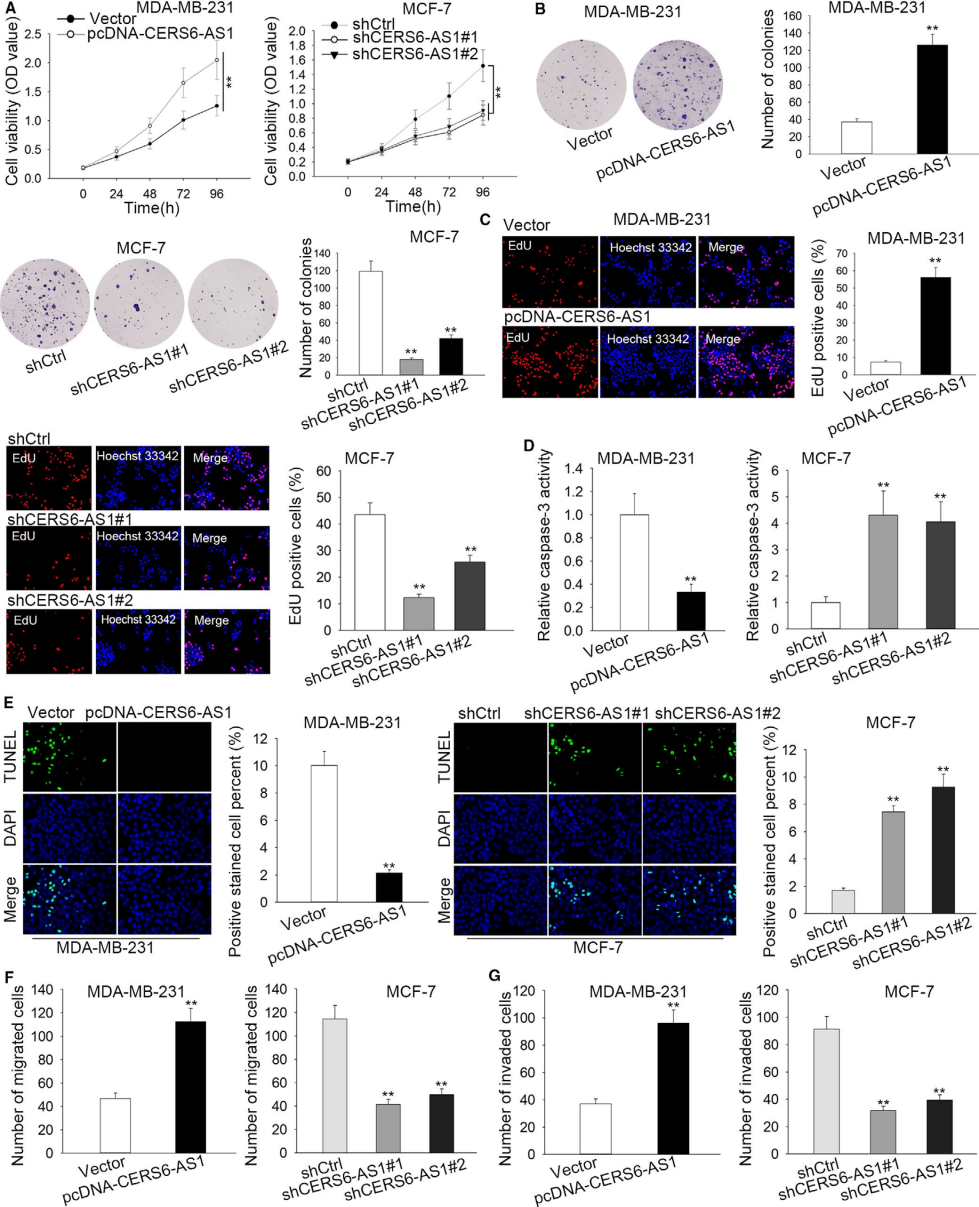
CERS6‐AS1 promotes proliferation and inhibits apoptosis in BC cells in vitro. A, The proliferation ability of transfected cells was researched by CCK8 assay. B, Colony formation assay detected cell proliferation. C, EdU assay presented the proliferation ability of transfected cells. D and E, Apoptosis ability of transfected cells was investigated by caspase‐3 activity and TUNEL assays. F and G, Cell migration and invasion were reflected by transwell assays. **P < .01

For the exploration of the biological role of CERS6‐AS1 in the development of BC, we upregulated CERS6‐AS1 through utilizing pcDNA‐CERS6‐AS1 with vector as scramble control and knocked down CERS6‐AS1 through using shCERS6‐AS1#1, shCERS6‐AS1#2, shCERS6‐AS1#3 with shCtrl as scramble control. Then, the upregulation and knockdown efficiencies were, respectively, detected in MDA‐MB‐231 and MCF‐7 cells by RT‐qPCR assay. As depicted in Figure S1A, the introduction of pcDNA‐CERS6‐AS1 caused a significant increase of CERS6‐AS1 levels in MDA‐MB‐231 cells in comparison with scramble control, indicating that pcDNA‐CERS6‐AS1 had the potential of being employed for the following gain‐of‐function assays. And the introduction of shCERS6‐AS1#1/2/3 conspicuously reduced CERS6‐AS1 expression in MCF‐7 cells compared with scramble control, of which shCERS6‐AS1#1 and shCERS6‐AS1#2 possessing the highest knockdown efficiency, therefore, we would use these two transfected cells in the following loss‐of‐function assays. To move on, the influences of CERS6‐AS1 overexpression and knockdown on cell proliferation and apoptosis were respectively evaluated in MDA‐MB‐231 and MCF‐7 cells. CCK8 assay suggested that cell proliferation was notably promoted in CERS6‐AS1‐upregulated MDA‐MB‐231 cells than that in control and decreased in CERS6‐AS1‐depleted MCF‐7 cells than that in control (Figure 2A). Colony formation assay revealed that CERS6‐AS1 upregulation induced a conspicuous increase in colony numbers in MDA‐MB‐231 cells and CERS6‐AS1 downregulation triggered a notable decrease in colony numbers in MCF‐7 cells (Figure 2B). EdU assay indicated that cell proliferation was notably increased in CERS6‐AS1‐upregulated MDA‐MB‐231 cells than that in control and decreased in CERS6‐AS1‐depleted MCF‐7 cells than that in control (Figure 2C). Caspase‐3 activity and TUNEL assays confirmed apoptosis ability was remarkably inhibited in CERS6‐AS1‐upregulated MDA‐MB‐231 cells and promoted in CERS6‐AS1‐depleted MCF‐7 cells than that in control (Figure 2D,E). Furthermore, transwell assays indicated that cell migration and invasion were facilitated by CERS6‐AS1 overexpression and impaired by CERS6‐AS1 deficiency (Figure 2F,G).

### 3.3 CERS6‐AS1 positively modulates the expression of CERS6

**Figure 3:**
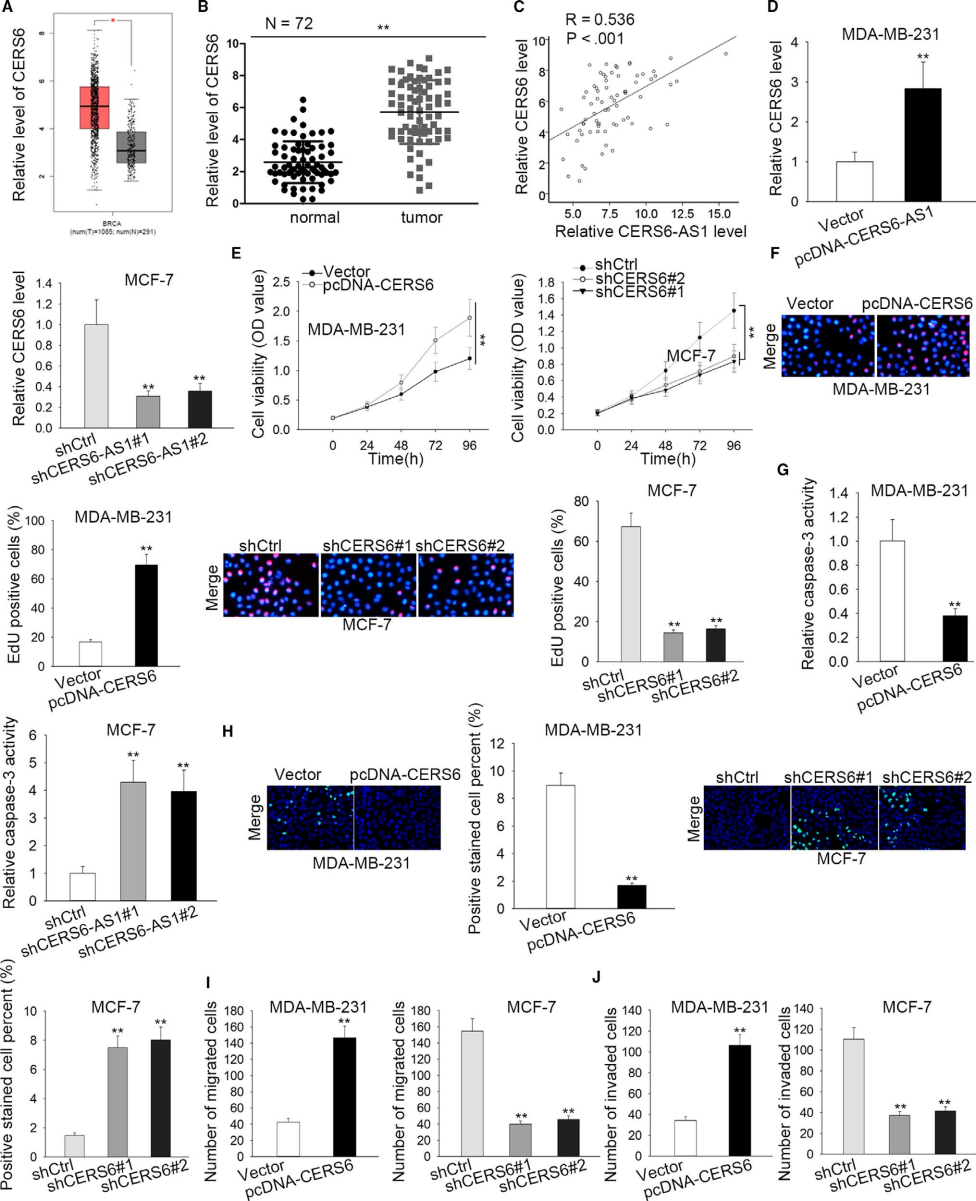
CERS6‐AS1 positively modulates the expression of CERS6. A, The expression of CERS6 in BC tissues (n = 1085) was higher than that in contiguous normal tissues (n = 291) in TCGA database. B, RT‐qPCR results revealed that CERS6‐AS1 expression was higher in tumor tissues than in contiguous normal tissues (n = 72). C, RT‐qPCR analysis revealed that expression of CERS6‐AS1 was positively related with that of CERS6 in tumor tissues. D, RT‐qPCR assay quantified the CERS6 expression in transfected cells. E and F, The proliferation ability of transfected cells was uncovered by CCK8 and EdU assays. G and H, Apoptosis ability of transfected cells was unveiled by Caspase‐3 activity and TUNEL assays. F and G, Transwell assays were carried out to evaluate cell migration and invasion. I, Number of migrated cells in CERS6‐overexpressed MDA‐MB‐231 cell or CERS6‐silenced MCF‐7 cell. J, Number of invaded cells in indicated BC cells after transfections. **P < .01

CERS6 is a nearby gene of CERS6‐AS1. To explore the roles of CERS6 in BC, we obtained its expression in BC tissues and contiguous normal tissues from TCGA database (1085 tumor tissues and 291 normal tissues). The result showed that CERS6 was conspicuously overexpressed in BC tissues than in contiguous normal tissues (Figure 3A). To further confirm the upregulation of CERS6 expression in BC, CERS6 expression in 72 paired BC tissues and contiguous normal tissues were measured. The result bears no difference with the above‐mentioned one: CERS6 was notably overexpressed in BC tissues (Figure 3B). It was suggested that CERS6 expression positively correlated with CERS6‐AS1 expression in BC tissues (Figure 3C). RT‐qPCR assay examined that CERS6 expression was conspicuously upregulated in CERS6‐AS1‐upregulated MDA‐MB‐231 cells than that in control and down‐regulated in CERS6‐AS1‐depleted MCF‐7 cells than that in control (Figure 3D), which suggests that CERS6‐AS1 positively modulates the expression of CERS6. For the further investigation of the possible biological part of CERS6 plays in BC tumorigenesis, we upregulated CERS6 through using pcDNA‐CERS6 with vector as scramble control and knocked down CERS6 through using shCERS6#1, shCERS6#2, shCERS6#3 with shCtrl as scramble control. As displayed in Figure S1B, the transfection of pcDNA‐CERS6 induced a conspicuous increase of CERS6 levels in MDA‐MB‐231 cells than in scramble control. And the introduction of shCERS6#1/2/3 caused a significant reduction of CERS6 levels in MCF‐7 cells than in scramble control. Next, the influences of CERS6 overexpression and knockdown on cell proliferation and apoptosis were, respectively, evaluated in MDA‐MB‐231 and MCF‐7 cells. CCK8 and EdU assays suggested that cell proliferation was notably increased in CERS6‐upregulated MDA‐MB‐231 cells than that in control and decreased in CERS6‐depleted MCF‐7 cells than that in control (Figure 3E,F). Caspase‐3 activity and TUNEL assays confirmed cell apoptosis were remarkably decreased in CERS6‐upregulated MDA‐MB‐231 cells than that in control and increased in CERS6‐depleted MCF‐7 cells than that in control (Figure 3G,H). In the end, as confirmed by transwell assay, number of migrated or invaded cells was increased by enforced expression of CERS6 and lowered by CERS6 depletion (Figure 3I,J). Taken together, CERS6 is overexpressed in BC cells, promoting the progression of BC, and is positively modulated by CERS6‐AS1.

Finally, as depicted in Figure S1C‐H, there is no notable difference between mock group, shCtrl group (or Vector group) concerning cell proliferation, apoptosis and invasion. Moreover, alteration of expression of CERS6‐AS1 or CERS6 contributed to the changes in cell proliferation, apoptosis and invasion. These results helped to rule out the influence of shRNA and plasmid.

### 3.4 IGF2BP3 serves as a RNA‐binding protein for CERS6‐AS1

**Figure 4:**
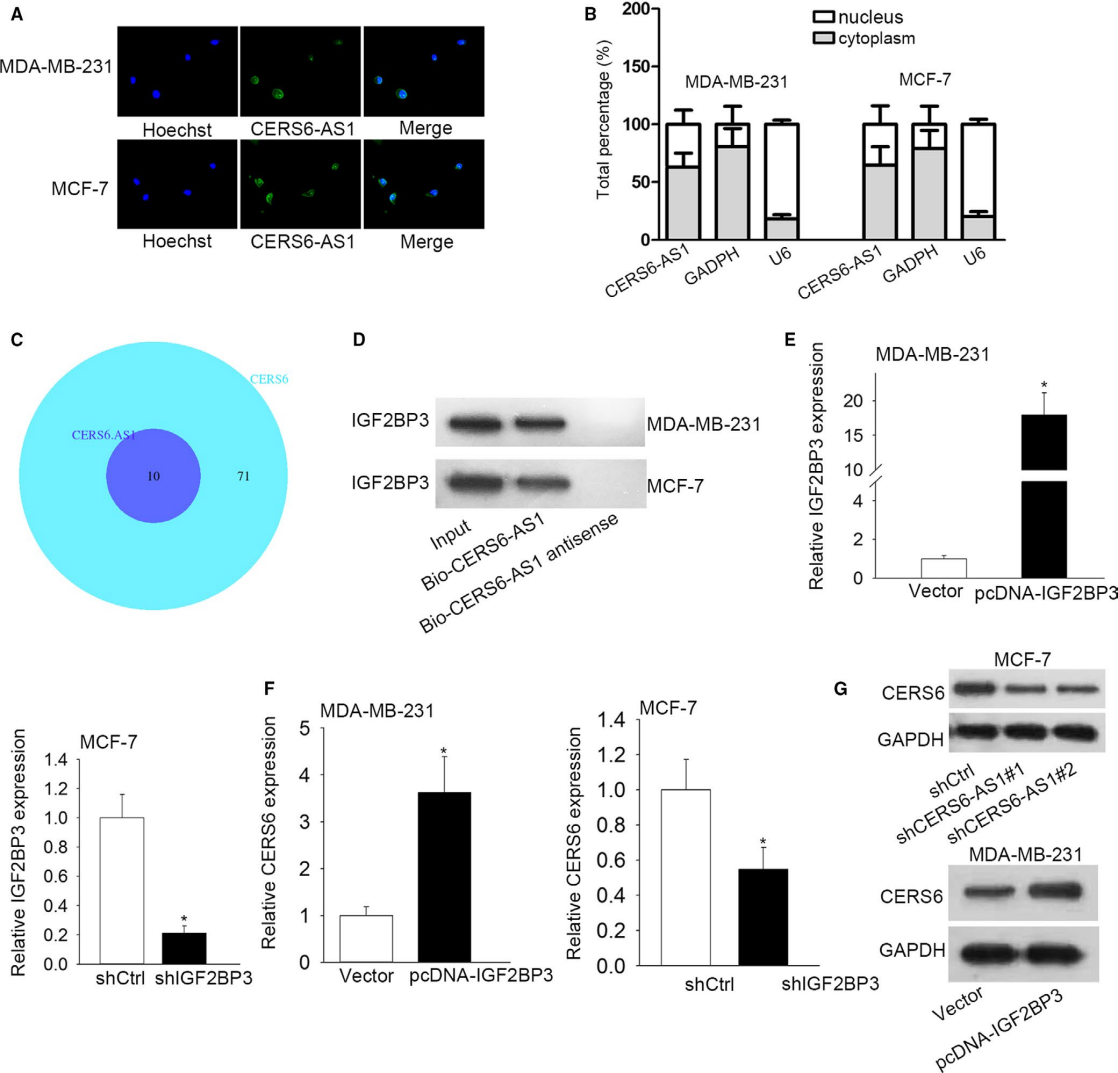
IGF2BP3 acts as a RBP of CERS6‐AS1 in BC. A, RNA FISH analysis was employed to detect the location of CERS6‐AS1 in BC cells. B, Subcellular fractionation assay analyzed the distribution of CERS6‐AS1 in nuclear and cytoplasm in BC cells. C, starBase website was utilized to screen out RBPs that may bind with both CERS6‐AS1 and CERS6. D, RNA pull down assay was applied to confirm the affinity between CERS6‐AS1 and IGF2BP3. E, The overexpression efficiency of IGF2BP3 in MDA‐MB‐231 cells and knockdown efficiency of IGF2BP3 in MCF‐7 cells were examined respectively by RT‐qPCR. F, RT‐qPCR assay analyzed the expression of CERS6 in transfected cells. G, CERS6 protein was detected in MCF‐7 cells transfected with shCtrl or sh‐CERS6‐AS1#1/#2 or MDA‐MB‐231 cells transfected with vector or pcDNA‐CERS6‐AS1. *P < .05

RNA FISH indicated that CERS6‐AS1 localized in cytoplasm (Figure 4A). Subcellular fractionation experiment manifested that CERS6‐AS1 largely located in cytoplasm (Figure 4B), which is consistent with the former conclusion. Recently, plenty of researches indicated that certain lncRNAs are implicated in regulating signaling pathways via the interaction with specific proteins.^14^, ^15^ starBase website was utilized and screened 10 RNA binding proteins (RBPs) that may bind with both CERS6‐AS1 and CERS6 (Figure 4C). It has been established that insulin‐like growth factor 2 mRNA‐binding protein 3 (IGF2BP3) is a RNA binding protein (RBP) that functions in cancers,^16^, ^17^ therefore, we hypothesized that IGF2BP3 may bind to CERS6‐AS1 in BC cells. RNA pull‐down was subsequently performed and the results showed that IGF2BP3 could only be pulled down by biotinylated CERS6‐AS1, suggesting that CERS6‐AS1 indeed bind with IGF2BP3 (Figure 4D). Additionally, we assessed the effect of CERS6‐AS1 on the protein level or stability of IGF2BP3. However, no significant changes were observed (Figure S2A,B), indicating CERS6‐AS1 might exert function by recruiting IGF2BP3. For further investigation of the underlying biological role of IGF2BP3 in BC tumorigenesis, we upregulated IGF2BP3 through using pcDNA‐IGF2BP3 with vector as scramble control and knocked down IGF2BP3 through using shIGF2BP3 with shCtrl as scramble control. Then, the upregulation and knockdown efficiency were, respectively, detected in MDA‐MB‐231 and MCF‐7 cells by RT‐qPCR assay. As shown in Figure 4E, the introduction of pcDNA‐IGF2BP3 caused an evident increase of IGF2BP3 levels in MDA‐MB‐231 cells than in scramble control, indicating that pcDNA‐IGF2BP3 had the possibility of being applied in the subsequent gain‐of‐function assays. And the introduction of shIGF2BP3 induced a significant reduction of IGF2BP3 levels in MCF‐7 cells than in scramble control, indicating that pcDNA‐IGF2BP3 possessed the potential of being employed for the coming loss‐of‐function assays. RT‐qPCR assay disclosed that CERS6 expression was considerably increased in IGF2BP3‐upregulated MDA‐MB‐231 cells and decreased in IGF2BP3‐depleted MCF‐7 cells (Figure 4F), which suggests that IGF2BP3 positively modulates the expression of CERS6. Moreover, we uncovered that CERS6‐AS1 positively regulated the protein level of CERS6 in BC cells (Figure 4G). To sum up, CERS6‐AS1 regulated CERS6 expression potentially by recruiting IGF2BP3 to stabilize CERS6.

### 3.5 CERS6‐AS1 promotes CERS6 mRNA stability by binding to IGF2BP3

**Figure 5:**
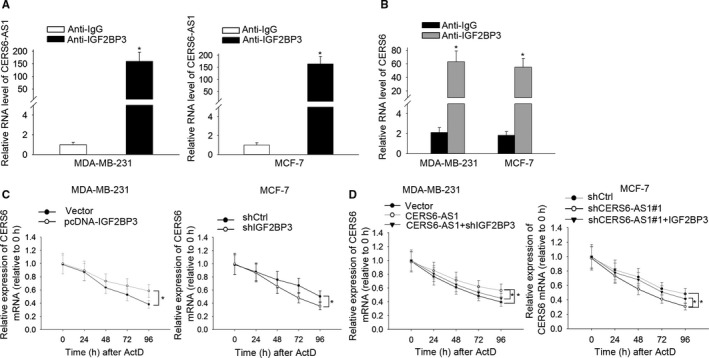
CERS6‐AS1 promotes the maintenance of CERS6 mRNA stability by binding to IGF2BP3. A and B, RIP and RT‐qPCR assays were conducted to examine the enrichment degrees of CERS6‐AS1 and CERS6 in IgG or IGF2BP3 immunoprecipitate. C, The rates of degradation of CERS6 mRNA in transfected cells were measured by RT‐qPCR. D, The rates of degradation of CERS6 mRNA in transfected cells were evaluated by RT‐qPCR. *P < .05

In order to explore the interaction among CERS6‐AS1, CERS6 and IGF2BP3, we conducted RIP assay and the results indicated that CERS6‐AS1 could bind with IGF2BP3 (Figure 5A) and CERS6 does so (Figure 5B). Considering the previous findings, it was inferred that CERS6‐AS1 may influence CERS6 expression via regulating CERS6 mRNA stability in BC. We discovered that IGF2BP3 overexpression upregulated CERS6 mRNA expression after treating with actinomycin D (ActD, a transcriptional inhibitor).

The results indicated that CERS6 mRNA stability increased in IGF2BP3‐upregulated MDA‐MB‐231 cells and decreased in IGF2BP3‐depleted MCF‐7 cells (Figure 5C). Furthermore, compared with pcDNA‐CERS6‐AS1 transfected MDA‐MB‐231 cells, CERS6 expression presented a decrease in pcDNA‐CERS6‐AS1 and sh‐IGF2BP3 co‐transfected MDA‐MB‐231 cells. In line, in comparison with sh‐CERS6‐AS1#1‐transfected MCF‐7 cells, CERS6 expression demonstrated recuperation in sh‐CERS6‐AS1#1 and pcDNA‐IGF2BP3 co‐transfected MCF‐7 cells (Figure 5D). It can be concluded that IGF2BP3 upregulation could partially rescue CERS6‐AS1 knockdown‐mediated decrease of CERS6 mRNA stability after treating with ActD. These evidences indicate that CERS6‐AS1 promotes CERS6 mRNA stability by binding to IGF2BP3.

### 3.6 Overexpression of CERS6 rescues the inhibition of CERS6‐AS1 deficiency on BC progression in vitro and vivo

**Figure 6:**
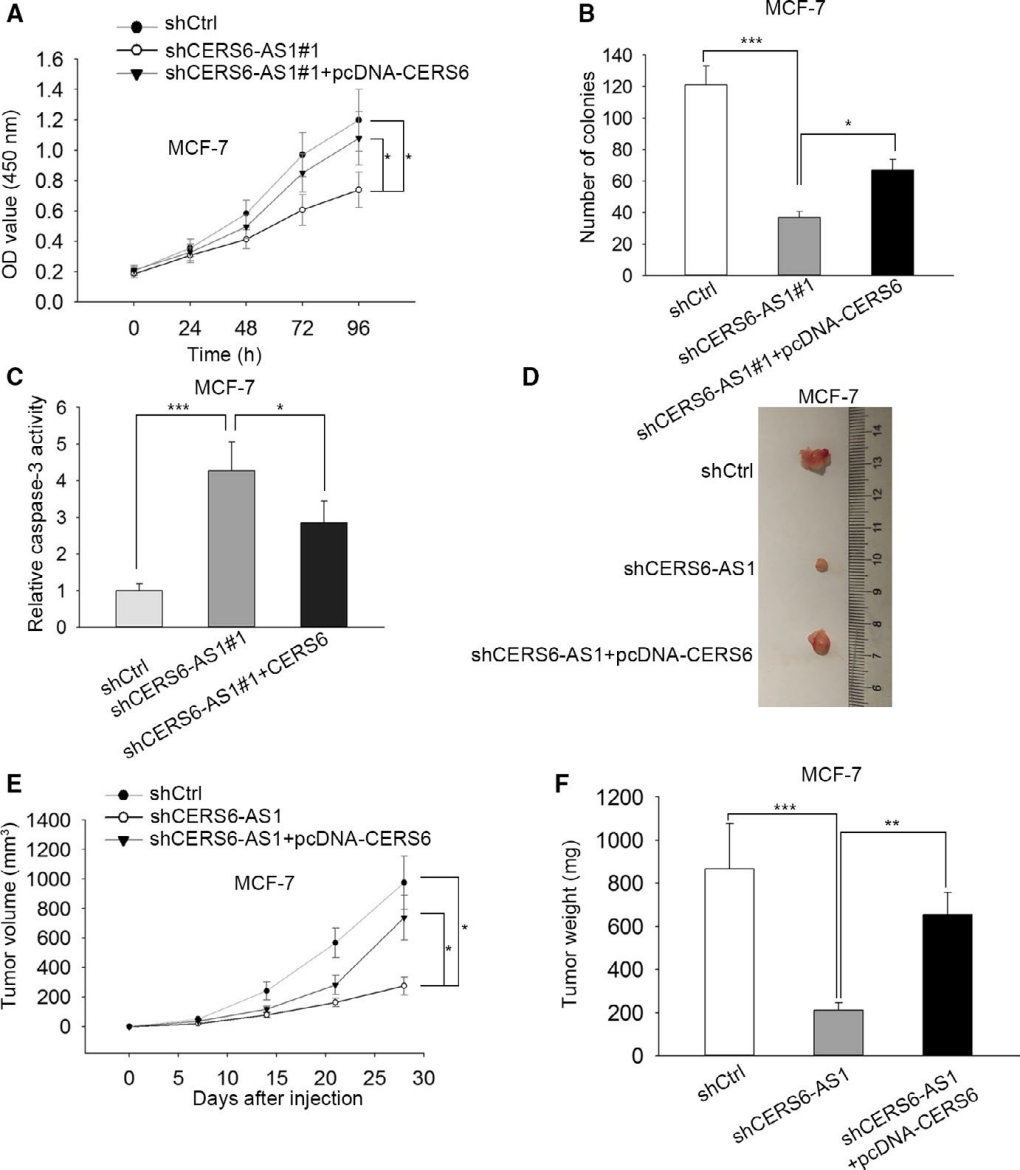
Overexpression of CERS6 reverses CERS6‐AS1 silencing‐mediated inhibitive effect on BC progression in vivo. A and B, CCK8 and colony formation implied that enforced expression of CERS6 rescues CERS6‐AS1 silencing‐mediated inhibitive effect on cell proliferation to some extent. C, Caspase‐3 activity assay implied that CERS6 overexpression rescues CERS6‐AS1 silencing‐mediated promotion of cell apoptosis to some degree. D, Approximately 4 weeks later, the tumors derived from transfected MCF‐7 cells were anatomized and photographed (n = 5 per group). E, The growth situation of CERS6‐AS1 silencing tumors in comparison with shCERS6‐AS1#1 and pcDNA‐CERS6 co‐transfected group were reflected in the graph. F, After the tumors were removed, tumor weights were measured. *P < .05, **P < .01, ***P < .001

To continue, we investigated whether CERS6‐AS1 promotes BC progression by targeting CERS6. CCK8 and colony formation results verified that enforced expression of CERS6 rescued cell proliferation of MCF‐7 cells which was decreased by CERS6‐AS1 knockdown (Figure 6A,B). Furthermore, Caspase‐3 activity assay indicated that the increase of cell apoptosis ability induced by CERS6‐AS1 knockdown was abrogated by CERS6 upregulation in MCF‐7 cells (Figure 6C). We continued exploring the role of CERS6 in vivo. MCF‐7 cells stably transfected sh‐CERS6‐AS1 or shCtrl were constructed for in vivo experiment (Figure S2C). It was discovered that the decreased tumor size, volume and weight of sh‐CERS6‐AS1#1‐transfected MCF‐7 cells were partially rescued by overexpression of CERS6 (Figure 6D,F). Taken together, overexpression of CERS6 rescues the inhibition of CERS6‐AS1 deficiency on BC progression in vitro and vivo.

## 4 DISCUSSION

Mounting evidence has testified that dysregulation of lncRNAs frequently occurs in the formation and development of various tumors such as breast cancer, cervical cancer and colorectal cancer.^18^, ^19^, ^20^ Nevertheless, the biological function and underlying molecular mechanism of CERS6‐AS1 in BC have not been clarified yet. In this study, it was detected that CERS6‐AS1 expression in BC tumor tissues and cells were considerably upregulated. CERS6‐AS1 knockdown suppressed the proliferation while promoted the apoptosis in BC cells. All these findings proofed that CERS6‐AS1 accelerates the progression of BC.

CER is not only an element of the membrane structure, but also a critical mediator of cellular functions, such as proliferation and apoptosis.^21^, ^22^, ^23^, ^24^ Disturbances regarding CERS and signaling have been discovered to be implicated in many sorts of cancers.^22^, ^25^, ^26^ As an influential member of the CERS family, dozens of studies have indicated that CERS6 is involved in cancers. For instance, CERS6 serves as an oncogene to promote the progression of gastric cancer through the SOCS2/JAK2/STAT3 signaling pathway.^27^ It is also reported that elevated CERS6 expression was associated with increased invasion of lung cancer cells and poor prognosis,^28^ while the function of CERS6 in BC remain unclear. Our study illustrated that CERS6 was upregulated in BC tumor tissues and cells. Absence of CERS6 impaired the proliferation while promoted the apoptosis in BC cells. What's more, CERS6‐AS1 positively modulated the expression of CERS6.

RBPs are proteins that bind to the double or single stranded RNA in cells. The stability of mRNA is regulated by thousands of RBPs.^29^ Besides, researches also showed that lncRNAs influence the development of various cancers through the interaction with RBPs.^30^, ^31^ It has been widely observed that dysregulation of these RBPs can lead to abnormal expression of cancer‐related genes.^32^ Previous studies have elucidated that IGF2BP3 exerts its oncogenic function in various cancer‐related processes.^33^, ^34^ In our current study, it was discovered that IGF2BP3 indeed served as a RBP for CERS6‐AS1 and CERS6‐AS1 promotes CERS6 mRNA stability by binding to IGF2BP3. Moreover, rescue assays indicated that upregulation of CERS6 countervailed CERS6‐AS1 knockdown‐mediated suppression of BC progression to some extent in vitro and vivo.

To put it in a nut shell, our paper elucidated that CERS6‐AS1 functions as a malignancy promoter in breast cancer by binding to IGF2BP3 to enhance the stability of CERS6 mRNA, implying that CERS6‐AS1 might serve as a molecular target for BC to improve prognosis.

## CONFLICT OF INTEREST

The authors declare that no competing interest exists in this study.

## ETHICS STATEMENT

All patients signed printed informed consent. All of the human participants or animals performed were approved by the Ethics Committee of the author's hospital.
