# Supplementary figures and images for: Long noncoding RNA CERS6‐AS1 functions as a malignancy promoter in breast cancer by binding to IGF2BP3 to enhance the stability of CERS6 mRNA
Source: Cancer Med. 2019 Nov 8;9(1):278–89. doi: 10.1002/cam4.2675 (PMC6943159; doi:10.1002/cam4.2675)

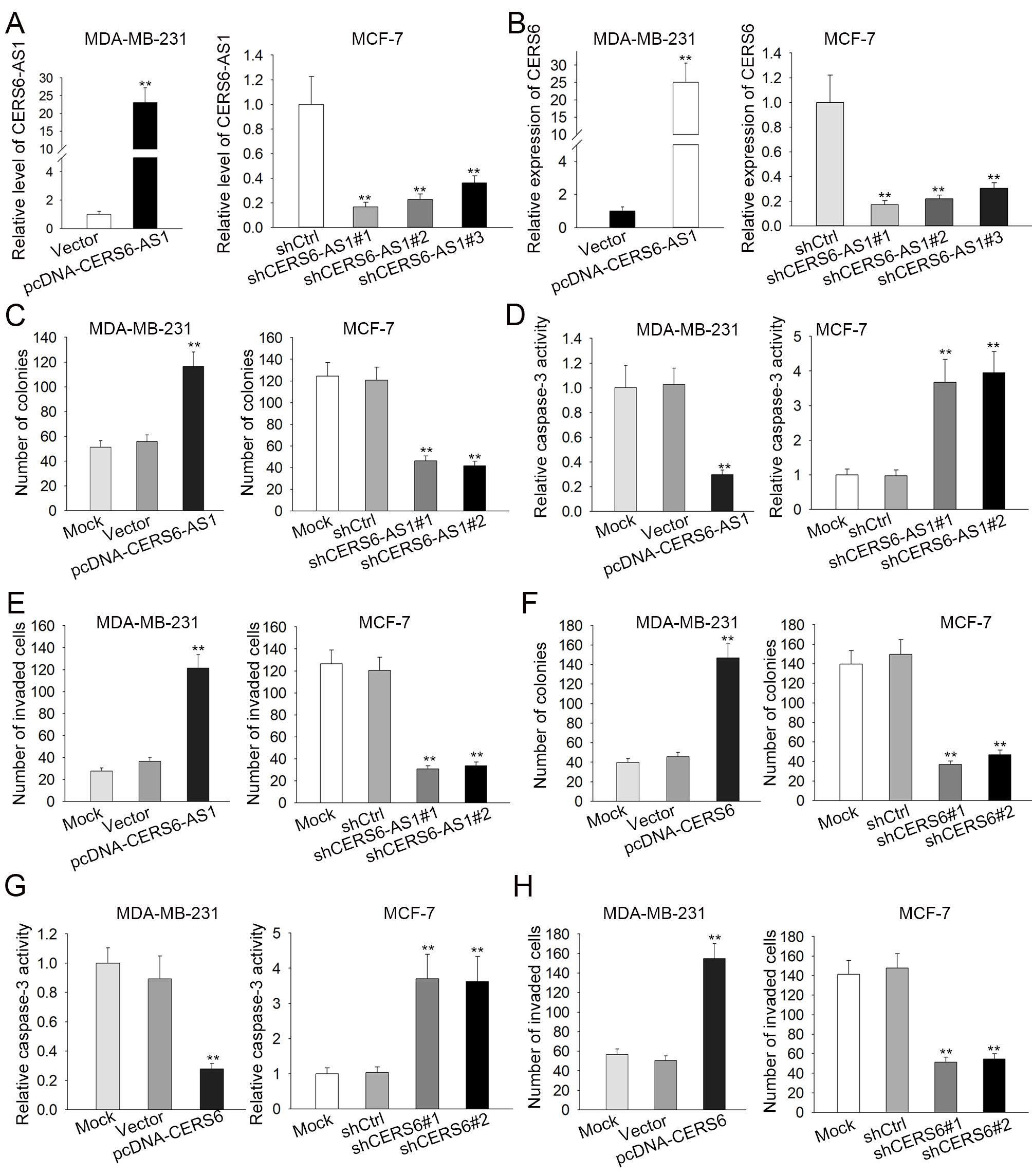

Supplement: Supplementary file 1 [file CAM4-9-278-s001.tif]

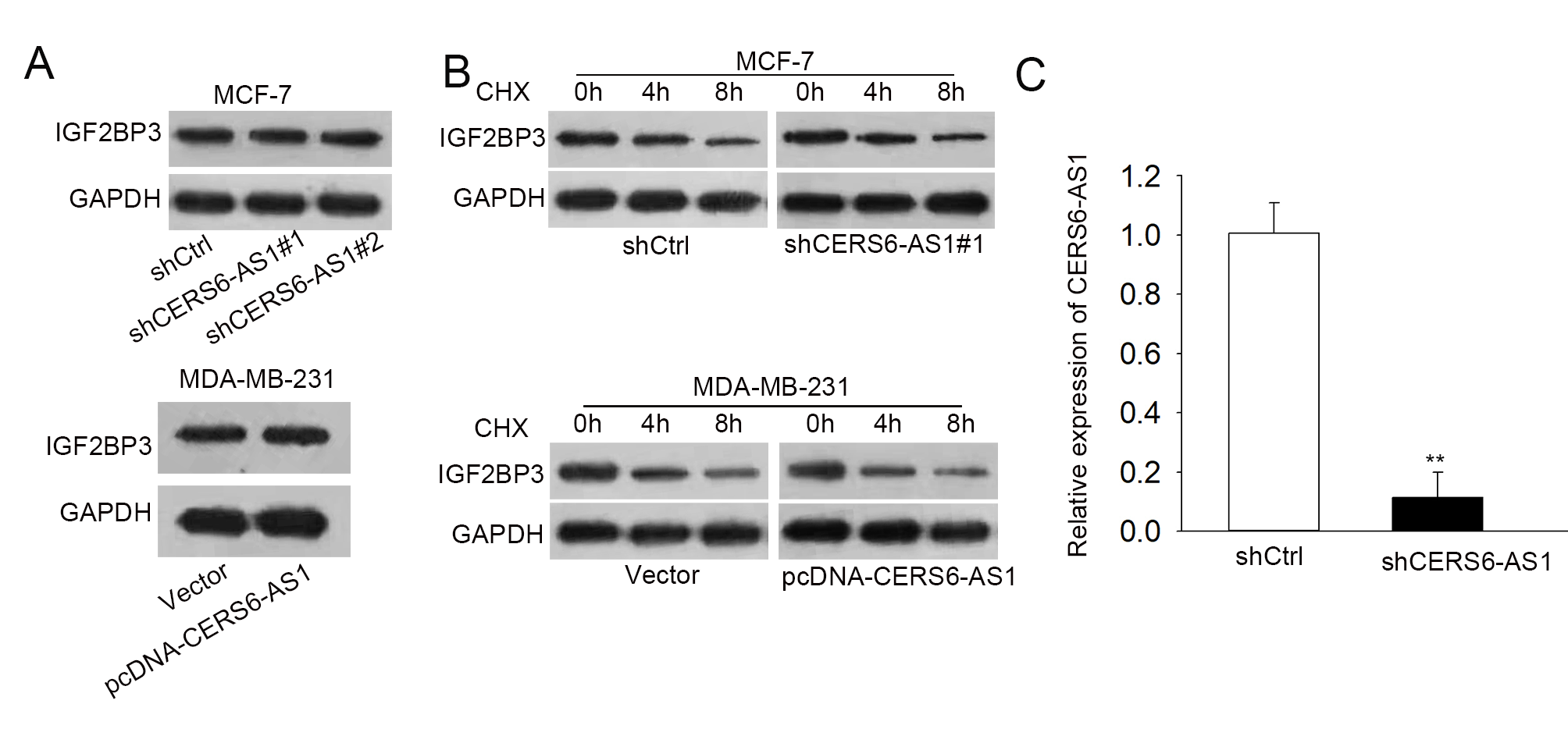

Supplement: Supplementary file 2 [file CAM4-9-278-s002.tif]
